# Optimal management of malignant left-sided large bowel obstruction: do international guidelines agree?

**DOI:** 10.1186/s13017-019-0242-5

**Published:** 2019-05-22

**Authors:** Peter John Webster, Joanna Aldoori, Dermot Anthony Burke

**Affiliations:** 1 Department of Colorectal Surgery, Leeds Teaching Hospitals NHS Trust, Beckett Street, Leeds, LS9 7TF UK; 2 Department of Colorectal Surgery, Hull and East Yorkshire NHS Hospitals Trust, Anlaby Road, Hull, HU3 2JZ UK

**Keywords:** Colorectal cancer, Bowel obstruction, Stent, Bridge to surgery

## Abstract

**Background:**

Approximately 20% of patients diagnosed with colorectal cancer will present with left-sided large bowel obstruction. The optimal management of this cohort of patients remains unclear. We aimed to review international guidelines to see if there was a consensus on the treatment of this surgical emergency.

**Methods:**

The PubMed and Medline databases were searched for guidelines on the management of left-sided, malignant large bowel obstruction (MBO) between 2010 and 2018.

**Results:**

Nineteen guidelines were identified spanning a range of continents. There was no clear consensus on the management of potentially resectable disease. Eight guidelines (42%) suggested primary surgery, two guidelines (11%) suggested stenting as a bridge to surgery and nine guidelines (47%) suggested surgery or stenting could be performed. Primary resection with or without anastomosis was the most frequently recommended procedure (*n* = 6 35%), but over a third of guidelines gave no operative recommendations. There was very limited detail on the stenting procedure and how long elective surgery should be deferred. In the palliative situation, there was general agreement that stents should be offered in preference to surgery.

**Conclusion:**

International guidelines offer limited and contrasting recommendations on the management of left-sided MBO. There is a lack of high-quality evidence to support whether emergency surgery or stenting as a bridge to surgery is the optimal procedure in terms of morbidity, mortality and long-term oncological outcome.

## Background

Colorectal cancer remains the most common cause of large bowel obstruction in adults [1], and around 20% of patients with colorectal cancer will present with this surgical emergency [2]. For obstructing right-sided colon cancers, there is a general consensus that primary resection and ileocolic anastomosis is the treatment of choice [3]. However, the most common site for malignant large bowel obstruction (MBO) is the sigmoid colon, and over 75% of obstructing cancers occur distal to the splenic flexure [4]. The optimal management of left-sided MBO is less clear [5].

Several surgical options exist for left-sided MBO including primary resection (with or without anastomosis), subtotal colectomy (with or without anastomosis) or defunctioning ileostomy/colostomy with interval resection [4]. Unfortunately, emergency surgery is associated with a high rate of morbidity and mortality [6, 7]. This is due, in part, to this cohort of patients often being elderly, with multiple co-morbidities and reduced physiological function. Mortality rates for emergency surgery have been reported to be almost three times that of elective resections [7].

More recently, self-expanding colonic endoluminal stents have been successfully used as a non-invasive technique to relieve left-sided MBO [8]. This allows surgical resection to be performed on an elective rather than emergency basis. Stenting as a bridge to surgery has resulted in higher rates of primary anastomosis, reduced numbers of permanent stomas and fewer wound infections with no increase in mortality compared to emergency surgery [9, 10]. Concerns have been raised regarding oncological outcomes [11] and stent-related morbidity [12]. However, recently published results of the largest phase III randomised controlled trial comparing stenting as a bridge to surgery with emergency surgery for left-sided MBO has shown no difference in mortality at 1 year [13].

One third of patients who present with MBO will never undergo curative resection [14]. Traditionally, these patients would be offered a defunctioning stoma to relieve the obstruction. However, stenting is having an increasing role in the palliative setting. Evidence shows it to be safe and that it offers an improved quality of life compared with emergency stoma formation [15].

The aim of this study was to perform a comparative review of international guidelines to see if a consensus exists on the optimal management of left-sided MBO.

## Methodology

**Fig. 1:**
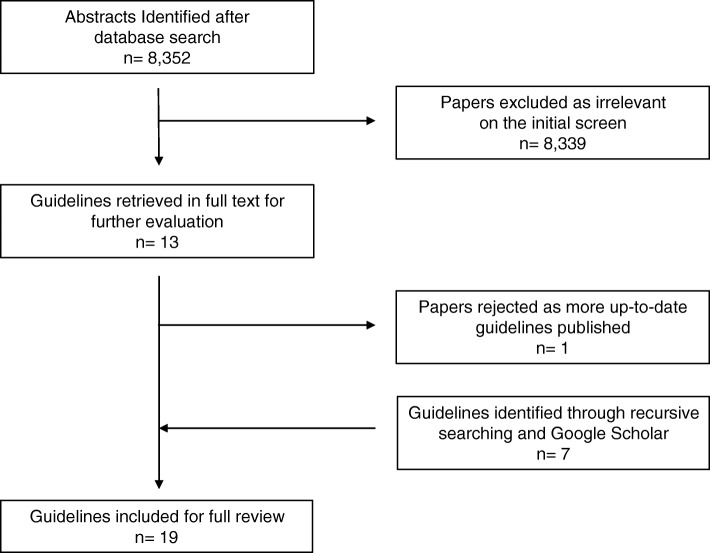
PRISMA diagram of the search strategy

As recommendations on managing MBO often form part of a more general guideline (e.g. management of colorectal cancer), our initial search strategy was broad. We searched the PubMed and Medline databases (2010–2018) using the Boolean operators [colon cancer OR colorectal cancer OR obstruction] AND [guideline*]. Inclusion criteria included any paper offering recommendations for the management of left-sided MBO in the English language. Initially retrieved articles were screened for relevance based on title, keywords and abstract review. Selected articles were then obtained in full text and reviewed by two independent reviewers (PJW, JA). Reference lists were recursively searched for further relevant articles. Google Scholar was also interrogated for any additional guidelines. A PRISMA diagram of the search strategy is provided in Fig. 1.

## Results

The search strategy returned 8352 citations. Of these, 8339 did not meet the inclusion criteria, leaving 13 articles for review. One article was rejected [2] as the guidelines from the publishing body had been updated and were included in the search [16]. Seven further articles were identified through a combination of recursive searching and Google Scholar. Therefore, 19 guidelines were included in the full review (Table 1) [16–34].

**Table 1:** Guidelines on the management of left-sided MBO

Year of publication	Origin of guidelines	Authors	Title of guidelines
2010	USA	American Society for Gastrointestinal Endoscopy (ASGE)	The role of endoscopy in the management of patients with known and suspected colonic obstruction and pseudo-obstruction [17]
2011	New Zealand	New Zealand Guidelines Group (NZGG)	Management of Early Colorectal Cancer [18]
2011	USA	National Comprehensive Cancer Network (NCCN)	Colon Cancer: Clinical Practice Guidelines in Oncology [19]
2013	Korea	Korean Society of Gastrointestinal Endoscopy	Evidence-Based Recommendations on Colorectal Stenting: A Report from the Stent Study Group of the Korean Society of Gastrointestinal Endoscopy [20]
2014	UK	National Institute for Health and Clinical Excellence (NICE)	Colorectal cancer: diagnosis and management (updated) [21]
2014	Europe	European Society of Gastrointestinal Endoscopy (ESGE)	Self-expandable metal stents for obstructing colonic and extracolonic cancer: Clinical Guideline [22]
2014	UK	Royal College of Surgeons of England (RCSEng)	Commissioning guide: Emergency general surgery (acute abdominal pain) [23]
2014	Germany	German Guideline Program in Oncology (GGPO)	Evidence-Based Guideline for Colorectal Cancer [24]
2014	Europe	European Registration of Cancer Care (EURECCA)	Multidisciplinary management: European consensus conference colon & rectum [25]
2014	France	French Society of Digestive Endoscopy (SFED) & French Federation of Digestive Oncology (FFCD)	Place of Colorectal Stents in Therapeutic Management of Malignant Large Bowel Obstructions [26]
2016	Scotland	Scottish Intercollegiate Guidelines Network (SIGN)	Diagnosis and Management of Colorectal Cancer (updated) [27]
2016	USA	Eastern Association for the Surgery of Trauma	Surgery or Stenting for Colonic Obstruction: A Practice Management Guideline from the Eastern Association for the Surgery of Trauma [28]
2016	Italy	Società Italiana di Chirurgia d’Urgenza e del Trauma (SICUT)	Clinical strategies for the management of intestinal obstruction and pseudo-obstruction [29]
2017	Malaysia	Malaysia Health Technology Assessment Section (MaHTAS)	Management of Colorectal Carcinoma [30]
2017	Australia	Cancer Council Australia	Emergency management of malignant large bowel obstruction [31]
2017	UK	British Medical Journal (BMJ) Best Practice	Large Bowel Obstruction [32]
2017	USA	American Society of Colon and Rectal Surgeons (ASCRS)	Clinical Practice Guidelines for the Treatment of Colon Cancer [33]
2017	UK	Association of Coloproctology of Great Britain & Ireland (ACPGBI)	Guidelines for the Management of Cancer of the Colon, Rectum and Anus (2017) - Surgical Management [34]
2018	Global	World Society of Emergency Surgery (WSES)	2017 WSES guidelines on colon and rectal cancer emergencies: obstruction and perforation [16]

### Nature of guidelines

**Fig. 2:**
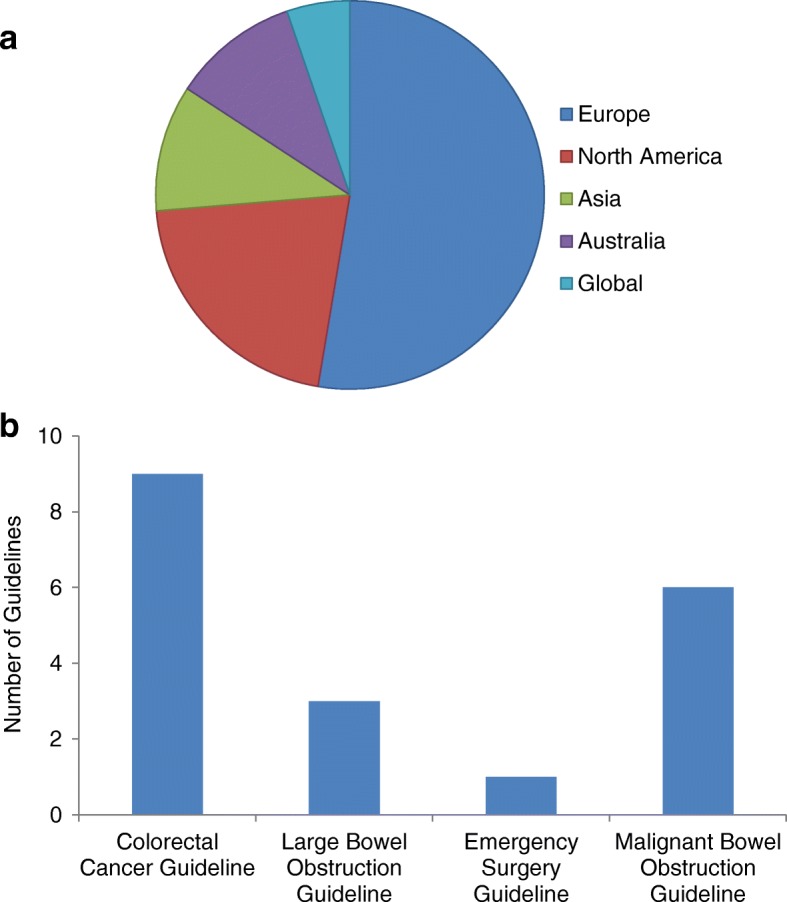
Properties of the reviewed guidelines. **a** Origin of the guidelines. **b** Context of the guidelines

The majority of guidelines originated from Europe (*n* = 10, 53%), with a smaller number from North America (*n* = 4, 21%), Australia (*n* = 2, 11%) and Asia (*n* = 2, 11%). One guideline was a global consensus (Fig. 2a). Most commonly, the guidelines formed part of a colorectal cancer guideline (*n* = 9, 47%) and less commonly a large bowel obstruction guideline (*n* = 3, 16%) or an emergency surgery guideline (*n* = 1, 5%). Six guidelines (32%) specifically focused on the management of MBO (Fig. 2b).

### Surgery

**Fig. 3:**
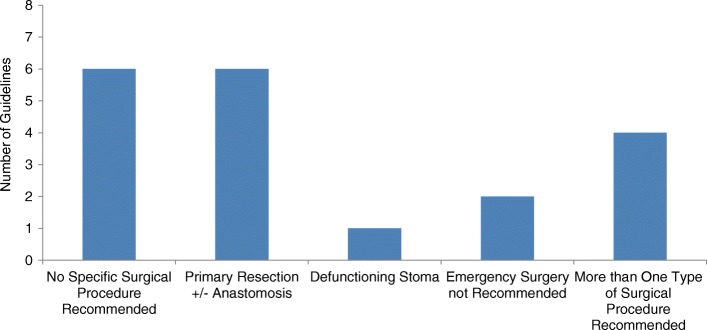
Recommended surgical procedures for the management of left-sided MBO

Eight of the guidelines (42%) recommended emergency surgery as the only treatment for left-sided MBO. A further nine guidelines (47%) recommended patients be offered emergency surgery or stenting as a bridge to surgery (Table 2). Of the 17 guidelines recommending emergency surgery, 6 (35%) recommended primary resection with or without anastomosis as the treatment of choice, one guideline (6%) recommended an emergency colostomy and 4 guidelines (24%) suggested multiple surgical options including defunctioning stoma (ileostomy, caecostomy, transverse loop colostomy, loop sigmoid colostomy), subtotal colectomy or primary resection (Fig. 3). Six guidelines (35%) gave no details regarding what operation should be performed. Only one guideline suggested that patients with left-sided MBO undergoing surgical intervention should be managed in an intensive care unit [34]. Two guidelines made reference to laparoscopic surgery; one guideline suggesting it had a limited role [23] and one guideline not recommending its use except in selected cases in specialist centres [16].

**Table 2:** Guideline recommendations on primary surgery or stenting as a bridge to elective surgery for the management of left-sided MBO

Authors	Title of guidelines	Surgery as primary treatment	Stenting as primary treatment	Level/quality of evidence
ASGE	The role of endoscopy in the management of patients with known and suspected colonic obstruction and pseudo-obstruction [17]	**X**	**✓**	Moderate
NZGG	Management of Early Colorectal Cancer [18]	**✓**	**✓**	III
NCNN	Colon Cancer: Clinical Practice Guidelines in Oncology [19]	**✓**	**✓**	NA
Korean Society of Gastrointestinal Endoscopy	Evidence-Based Recommendations on Colorectal Stenting: A Report from the Stent Study Group of the Korean Society of Gastrointestinal Endoscopy [20]	**✓**	**✓**	Moderate
NICE	Colorectal cancer: diagnosis and management (updated) [21]	**✓**	**✓**	Low
ESGE	Self-expandable metal stents for obstructing colonic and extracolonic cancer: Clinical Guideline [22]	**✓**	**X**	Low
RCSEng	Commissioning guide: Emergency general surgery (acute abdominal pain) [23]	**✓**	**✓**	NA
GGPO	Evidence-Based Guideline for Colorectal Cancer [24]	**✓**	**X**	NA
EURECCA	Multidisciplinary management: European consensus conference colon & rectum [25]	**✓**	**✓**	NA
SFED & FFCD	Place of Colorectal Stents in Therapeutic Management of Malignant Large Bowel Obstructions [26]	**✓**	**X**	IV
SIGN	Diagnosis and Management of Colorectal Cancer (updated) [27]	**✓**	**X**	II+
Eastern Association for the Surgery of Trauma	Surgery or Stenting for Colonic Obstruction: A Practice Management Guideline from the Eastern Association for the Surgery of Trauma [28]	**X**	**✓**	Low
SICUT	Clinical strategies for the management of intestinal obstruction and pseudo-obstruction [29]	**✓**	**X**	NA
MaHTAS	Management of Colorectal Carcinoma [30]	**✓**	**X**	I
Cancer Council Australia	Emergency management of malignant large bowel obstruction [31]	**✓**	**X**	II
BMJ	Large Bowel Obstruction [32]	**✓**	**✓**	NA
ASCRS	Clinical Practice Guidelines for the Treatment of Colon Cancer [33]	**✓**	**✓**	Moderate
ACPGBI	Guidelines for the Management of Cancer of the Colon, Rectum and Anus (2017) - Surgical Management [34]	**✓**	**✓**	II
WSES	2017 WSES guidelines on colon and rectal cancer emergencies: obstruction and perforation [16]	**✓**	**X**	IB

*NA* not attempted

### Stenting as a bridge to surgery

**Fig. 4:**
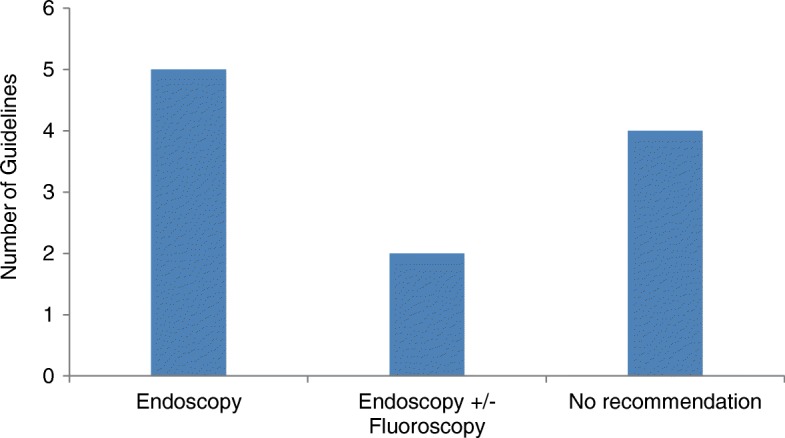
Recommended method of stenting

Two guidelines (11%) recommended emergency stenting as a bridge to elective surgery rather than primary surgery (Table 2). Both guidelines originated from America. In total, 11 guidelines (58%) advised stenting could be offered as the primary treatment. There was no consensus on whether stents should be inserted endoscopically, radiologically or by using a combination of the two techniques (Fig. 4). Also, there was limited guidance on the proximal limit of stenting. One guideline suggested the splenic flexure as a proximal limit [32]; however, another guideline recommended their use for obstructing right-sided colon cancers [33]. Equally, there were few recommendations about when surgery should be performed following stenting. One guideline suggested “within two weeks” [23], whereas a separate guideline, which did not recommend stenting as a bridge to surgery, recommended 5–10 days [22].

Eight guidelines (42%) did not recommend stenting as a bridge to surgery as the primary treatment for left-sided MBO (Table 2). Reasons against stenting included “no overall benefit compared to surgery” (*n* = 4), “risks of perforation” (*n* = 3) and “oncological concerns” (*n* = 3).

### Palliation

**Fig. 5:**
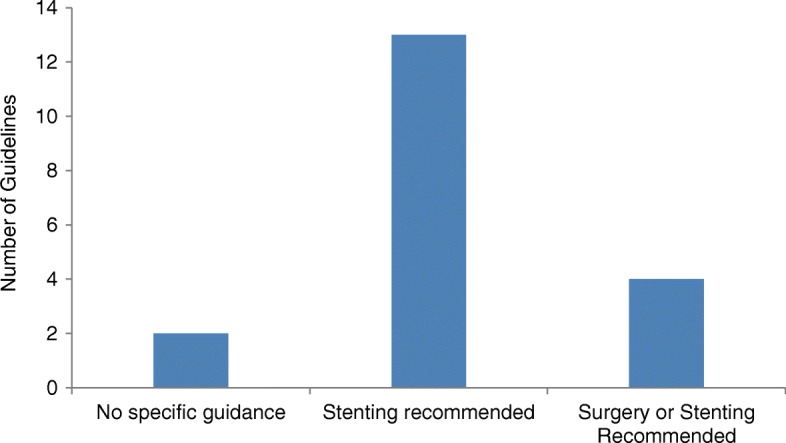
Recommendations for the management of palliative patients with left-sided MBO

Seventeen guidelines (89%) commented on the palliative management of left-sided MBO. The majority recommended stenting as the treatment of choice (*n* = 13, 76%). Four guidelines (24%) suggested stenting or surgery could be considered and no guidelines recommended surgery alone (Fig. 5). Recommended surgical options included primary resection with anastomosis, defunctioning stoma or bypass surgery. Five guidelines (29%) warned against the use of stents if anti-angiogenic agents were being considered, due to an increased risk of intestinal perforation.

### Quality of evidence

Of the 19 guidelines, 13 (68%) appraised the quality of evidence available to formulate their recommendations (Table 2). Several different tools were used to grade the evidence, but the most commonly used was the Grading of Recommendations Assessment, Development and Evaluation (GRADE) system [35]. Thirteen guidelines were published between 2010 and 2016. Most of these guidelines were based on low quality or level III–IV evidence (*n* = 5, 38%). In contrast, six guidelines were published between 2017 and 2018 and these were mostly based on moderate quality or level I–II evidence (*n* = 5, 83%). To date, no guidelines have reported on high quality or level Ia evidence.

## Discussion

This study has confirmed there is no clear consensus amongst international guidelines regarding the optimal management of resectable left-sided MBO. Most guidelines suggest that either primary surgery or stenting as a bridge to elective surgery can be offered to patients. High-quality evidence is lacking, however, to determine the best management strategy in terms of morbidity, mortality and long-term oncological outcomes. Furthermore, guidelines specific to the management of MBO are lacking. In this comparative review, most publications featured a few paragraphs on MBO as part of a wider guideline on colorectal cancer. This is surprising given that one in five patients with colorectal cancer will present this way.

Stenting for MBO is a relatively new treatment that was introduced in the 1990s, initially as a palliative procedure. Its role as a bridge to elective surgery soon developed and initial results were promising [36]. Subsequently, randomised controlled trials comparing stenting as a bridge to elective surgery against emergency surgery raised concerns regarding adverse outcomes and oncological efficacy [12, 37–39]. At this time, there was a clear shift in the guideline recommendations, with a number advising against stenting between 2014 and 2016 as demonstrated in Table 2. However, several more recent studies have reported that stenting does not compromise oncological outcomes [13, 40, 41] and more recent guidelines have begun advocating stenting as a bridge to surgery once again. Several guidelines were published well before the results of more recent randomised controlled trials [10, 13], and this may in part explain the lack of consensus on recommendations. It is clear to see that the quality of evidence upon which recommendations are made has improved over time; however, there remains a lack of high-quality evidence, particularly on long-term oncological outcomes. Contrasting recommendations based on the appraisal of different levels of evidence makes it difficult for surgeons to know which set of guidelines to follow and what is the optimal management strategy for this surgical emergency.

In the palliative setting, the guidelines are more consistent, recommending stenting as the preferred management option in most cases. However, it is somewhat unclear why guidelines that do not recommend stenting as a bridge to surgery due to fear of adverse outcomes (such as intestinal perforation) recommend stents in a palliative setting. Surely the risk of perforation in the palliative setting is equal? Similarly, if stenting has been shown to be safe in the palliative setting [15], why would there be an increased risk of perforation in those patients undergoing delayed elective resection? Naturally, the concern centres on converting a potentially resectable cancer to an unresectable cancer with stent-associated perforation. Nevertheless, in the ESCO trial, no difference in oncological outcome was reported at 3 years between the emergency surgery and stenting as a bridge to surgery groups [10]. The authors postulated that previously reported high rates of stent-associated perforation were due to variation in operator experience [10]. Clearly, more long-term data are necessary to confirm this, but at the very least this confirms that stenting as a bridge to surgery should only be considered in specialist centres with expertise in stenting procedures.

Guidelines that advocate stenting clearly lack detail. There are limited recommendations on technique, who should perform the stenting procedure, what is the proximal limit for stenting and when the optimal time to perform subsequent surgery is. This may reflect the fact that it is a relatively new technique and high-quality evidence is lacking for these recommendations. For instance, there are limited reports regarding the optimal interval for surgery following stenting. One small study reported reduced anastomotic leak rates when surgery was delayed for more than 10 days after stent insertion [42].

Emergency endoluminal colonic stenting is not available in all hospitals in the UK. Guidelines that advocate stenting as a bridge to surgery over emergency surgery make no comment about what to do if the hospital does not have provisions for stenting. Should these patients be transferred to specialist centres rather than undergo emergency surgery in their own institution? If a patient is admitted to a smaller district general hospital, they are far more likely to have emergency surgery. Historically, surgery has always been a treatment for MBO and although 17 guidelines (89%) recommended surgery, there was disagreement about which operation to perform. Primary resection, with or without anastomosis, was the most common recommendation, but over a third of guidelines did not recommend a specific operation. Clearly, there is no consensus amongst international guidelines regarding the management of resectable left-sided MBO. In reality, most groups continue to use a two-stage approach, either a Hartmann’s procedure or stenting in the emergency setting, followed by elective surgery [4].

## Conclusion

International guidelines offer limited and contrasting recommendations on the management of left-sided MBO. There is a lack of high-quality evidence to support whether emergency surgery or stenting as a bridge to surgery is the best procedure with regards to morbidity, mortality and long-term oncological outcomes. Comparison of the guidelines is difficult as they are based on differing levels of evidence and a number require updating to consider the results of more recent randomised controlled trials. Furthermore, a number of guidelines fail to provide a formal evaluation of the evidence at all. This review provides a snapshot of current guidelines, and until high-quality research is available, the optimal management of this emergency surgical condition will continue to be debated.

## Abbreviations

GRADE: Grading of Recommendations Assessment, Development and Evaluation
MBO: Malignant bowel obstruction
NA: Not attempted
